# (*E*)-1-(4-Amino­phen­yl)-3-(2,4,5-trimeth­oxy­phen­yl)prop-2-en-1-one

**DOI:** 10.1107/S1600536810026346

**Published:** 2010-07-10

**Authors:** Hoong-Kun Fun, Thawanrat Kobkeatthawin, Pumsak Ruanwas, Suchada Chantrapromma

**Affiliations:** a X-ray Crystallography Unit, School of Physics, Universiti Sains Malaysia, 11800 USM, Penang, Malaysia; b Crystal Materials Research Unit, Department of Chemistry, Faculty of Science, Prince of Songkla University, Hat-Yai, Songkhla 90112, Thailand

## Abstract

Mol­ecules of the title amino­chalcone, C_18_H_19_NO_4_, are twisted, with a dihedral angle of 11.26 (6)° between the 4-amino­phenyl and 2,4,5-trimeth­oxy­phenyl rings. The conformations of the three meth­oxy groups with respect to the benzene ring are slightly different. Two meth­oxy groups are almost coplanar with the attached benzene ring [C—O—C—C torsion angles of −1.45 (19) and 1.5 (2)°], while the third is (−)-synclinal with the attached benzene ring [C—O—C—C = −81.36 (17)°]. In the crystal structure, mol­ecules are stacked into columns along the *b* axis and mol­ecules in adjacent columns are linked by N—H⋯O hydrogen bonds into V-shaped double columns. Weak π–π inter­actions are also observed, with a centroid-centroid distance of 3.7532 (8) Å.

## Related literature

For bond-length data, see: Allen *et al.* (1987 ▶). For hydrogen bond motifs, see: Bernstein *et al.* (1995 ▶). For related structures, see: Chantrapromma *et al.* (2009 ▶, 2010 ▶); Suwunwong *et al.* (2009 ▶). For background to and applications of chalcones, see: Batovska *et al.* (2007 ▶); Jung *et al.* (2008 ▶); Kim *et al.* (2010 ▶); Nielsen *et al.* (2004 ▶); Niu *et al.* (2006 ▶); Romagnoli *et al.* (2008 ▶); Tewtrakul *et al.* (2003 ▶); Won *et al.* (2005 ▶); Xia *et al.* (2000 ▶).
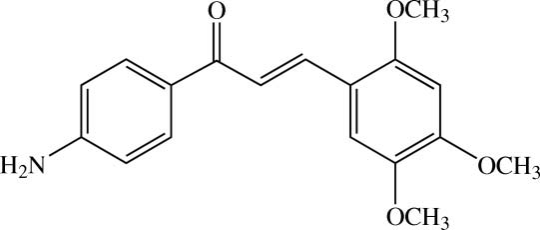


## Experimental

### 

#### Crystal data

C_18_H_19_NO_4_
*M* *_r_* = 313.34
Monoclinic, 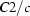

*a* = 13.6117 (2) Å
*b* = 10.3540 (2) Å
*c* = 22.3920 (4) Å
β = 100.879 (1)°
*V* = 3099.11 (9) Å^3^
*Z* = 8
Mo *K*α radiation
μ = 0.10 mm^−1^
*T* = 100 K
0.38 × 0.32 × 0.10 mm

#### Data collection

Bruker APEXII CCD area-detector diffractometer
Absorption correction: multi-scan (*SADABS*; Bruker, 2005 ▶) *T*_min_ = 0.965, *T*_max_ = 0.991
19818 measured reflections
4506 independent reflections
3581 reflections with *I* > 2σ(*I*)
*R*_int_ = 0.029

#### Refinement

*R*[*F* ^2^ > 2σ(*F* ^2^)] = 0.051
*wR*(*F* ^2^) = 0.131
*S* = 1.05
4506 reflections
219 parameters
H atoms treated by a mixture of independent and constrained refinement
Δρ_max_ = 0.43 e Å^−3^
Δρ_min_ = −0.23 e Å^−3^

### 

Data collection: *APEX2* (Bruker, 2005 ▶); cell refinement: *SAINT* (Bruker, 2005 ▶); data reduction: *SAINT*; program(s) used to solve structure: *SHELXTL* (Sheldrick, 2008 ▶); program(s) used to refine structure: *SHELXTL*; molecular graphics: *SHELXTL*; software used to prepare material for publication: *SHELXTL* and *PLATON* (Spek, 2009 ▶).

## Supplementary Material

Crystal structure: contains datablocks global, I. DOI: 10.1107/S1600536810026346/fj2326sup1.cif

Structure factors: contains datablocks I. DOI: 10.1107/S1600536810026346/fj2326Isup2.hkl

Additional supplementary materials:  crystallographic information; 3D view; checkCIF report

## Figures and Tables

**Table 1:** Hydrogen-bond geometry (Å, °)

*D*—H⋯*A*	*D*—H	H⋯*A*	*D*⋯*A*	*D*—H⋯*A*
N1—H1*N*1⋯O1^i^	0.86 (2)	2.12 (2)	2.9692 (16)	170.4 (17)
N1—H2*N*1⋯O1^ii^	0.88 (2)	2.21 (2)	3.0176 (17)	153.4 (19)

Symmetry codes: (i) 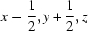; (ii) 
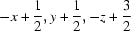
.

**Fig. 1.**
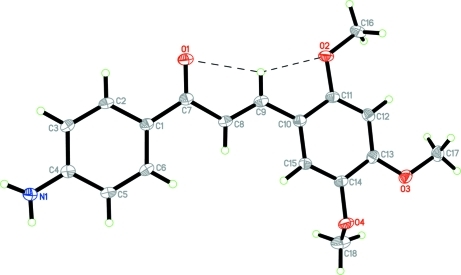
The molecular structure of the title compound, showing 50% probability displacement ellipsoids and the atom-numbering scheme. The dashed lines represent the intramolecular C—H···O interactions.

**Fig. 2.**
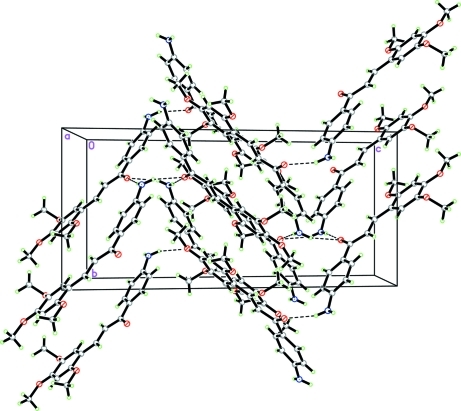
The crystal packing of the title compound viewed along the a axis. Hydrogen bonds are shown as dashed lines.

## supplementary crystallographic information

### Comment

Chalcones are important compounds which have considerable interesting applications involving biological activities such as antibacterial (Nielsen *et al.*, 2004), antifungal (Batovska *et al.*, 2007), anti-inflammatory (Won *et al.*, 2005), anti-HIV-1 protease inhibition (Tewtrakul *et al.*, 2003) and antitumor (Romagnoli *et al.*, 2008) properties. Moreover they also show electroactive fluorescent properties (Jung *et al.*, 2008; Niu *et al.*, 2006). From previous reports, aminochalcone showed good cytotoxicity in *vitro* against human tumor cell lines (Xia *et al.*, 2000) and was a new class of both activator and inhibitor of the TREK2 channel which turn out to be an important channel in charge of tuning neuronal transmitter and hormone levels (Kim *et al.*, 2010). In continuing our on-going research on bioactivity and fluorescence properties of chalcones (Chantrapromma *et al.*, 2009; 2010; Suwunwong *et al.*, 2009), the title aminochalcone (I) was synthesized. (I) doesn't possess antibacterial and cytotoxic activities. However (I) exhibits fluorescence with the maximum emission at 480 nm when the compound is excited at 320 nm in chloroform solution.

The molecule of the title aminochalcone, (Fig. 1), exists in an *E* configuration with respect to the C8═C9 double bond [1.3407 (19) Å] with torsion angle C7–C8–C9–C10 = 177.99 (13)°. The whole molecule is not planar indicated by the dihedral angle between 4-aminophenyl and 2,4,5-trimethoxyphenyl rings being 11.26 (6)°. The propenone unit (C7—C9/O1) is planar with the *r.m.s*. deviation 0.0031 (1) Å. The mean plane through the propenone unit makes dihedral angles of 11.70 (9) and 9.19 (9)° respectively with the planes of 4-aminophenyl and 2,4,5-trimethoxyphenyl rings. The three methoxy groups of 2,4,5-trimethoxyphenyl unit have two different orientations: two methoxy groups (at atom C11 and C13 positions) are co-planar with the attached benzene ring with torsion angles C16–O2–C11–C12 = -1.45 (19)° and C17–O3–C13–C12 = 1.5 (2)° whereas the one at atom C14 position is (-)-*syn*-clinally attached with the torsion angle C18–O4–C14–C13 = -81.36 (17)°. Weak C9—H9A···O1 and C9—H9A···O2 intramolecular interactions generate two S(5) motifs (Bernstein *et al.*, 1995) (Fig. 1 and Table 1). The bond distances and angles are of normal values (Allen *et al.*, 1987) and are comparable with the related structures (Chantrapromma *et al.*, 2009; 2010; Suwunwong *et al.*, 2009).

In the crystal packing (Fig. 2), the molecules are stacked into columns along the *b* axis. The molecules in the adjacent columns are linked by N—H···O hydrogen bonds (Table 1) into a V-shape double columns. π–π interaction was observed with the *Cg*_1_···*Cg*_2_ distance of 3.7532 (8) Å (symmetry code -1/2 - *x*, 3/2 - *y*, 1 - *z*); *Cg*_1_ and *Cg*_2_ are the centroids of C1–C6 and C10–C15 rings, respectively.

### Experimental

The title compound was synthesized by the condensation of 2,4,5-trimethoxybenzaldehyde (0.40 g, 2 mmol) with 4-aminoacetophenone (0.28 g, 2 mmol) in ethanol (30 ml) in the presence of 30% NaOH(aq) (5 ml). After stirring for 3 h at room temperature, the resulting orange solid appeared and was then collected by filtration, washed with distilled water, dried and purified by repeated recrystallization from acetone. Orange block-shaped single crystals of the title compound suitable for *x*-ray structure determination were recrystallized from acetone by the slow evaporation of the solvent at room temperature after several days, Mp. 393–394 K.

### Refinement

Amino H atoms were located in difference maps and refined isotropically. The remaining H atoms were positioned geometrically and allowed to ride on their parent atoms, with d(C—H) = 0.93 Å for aromatic and 0.96 Å for CH_3_ atoms. The *U*_iso_ values were constrained to be 1.5*U*_eq_ of the carrier atom for methyl H atoms and 1.2*U*_eq_ for the remaining H atoms. A rotating group model was used for the methyl groups. The highest residual electron density peak is located at 0.74 Å from H18C and the deepest hole is located at 1.39 Å from C15.

### Crystal data

**Table:**

C_18_H_19_NO_4_	*F*(000) = 1328
*M**_r_* = 313.34	*D*_x_ = 1.343 Mg m^−^^3^
Monoclinic, *C*2/*c*	Melting point = 393–394 K
Hall symbol: -C 2yc	Mo *K*α radiation, λ = 0.71073 Å
*a* = 13.6117 (2) Å	Cell parameters from 4506 reflections
*b* = 10.3540 (2) Å	θ = 1.9–30.0°
*c* = 22.3920 (4) Å	µ = 0.10 mm^−^^1^
β = 100.879 (1)°	*T* = 100 K
*V* = 3099.11 (9) Å^3^	Block, orange
*Z* = 8	0.38 × 0.32 × 0.10 mm

### Data collection

**Table:**

Bruker APEXII CCD area-detector diffractometer	4506 independent reflections
Radiation source: sealed tube	3581 reflections with *I* > 2σ(*I*)
graphite	*R*_int_ = 0.029
φ and ω scans	θ_max_ = 30.0°, θ_min_ = 1.9°
Absorption correction: multi-scan (*SADABS*; Bruker, 2005)	*h* = −19→19
*T*_min_ = 0.965, *T*_max_ = 0.991	*k* = −14→12
19818 measured reflections	*l* = −31→31

### Refinement

**Table:**

Refinement on *F*^2^	Primary atom site location: structure-invariant direct methods
Least-squares matrix: full	Secondary atom site location: difference Fourier map
*R*[*F*^2^ > 2σ(*F*^2^)] = 0.051	Hydrogen site location: inferred from neighbouring sites
*wR*(*F*^2^) = 0.131	H atoms treated by a mixture of independent and constrained refinement
*S* = 1.05	*w* = 1/[σ^2^(*F*_o_^2^) + (0.0552*P*)^2^ + 3.4363*P*] where *P* = (*F*_o_^2^ + 2*F*_c_^2^)/3
4506 reflections	(Δ/σ)_max_ < 0.001
219 parameters	Δρ_max_ = 0.43 e Å^−^^3^
0 restraints	Δρ_min_ = −0.23 e Å^−^^3^

### Special details

**Table:**

Experimental. The crystal was placed in the cold stream of an Oxford Cryosystems Cobra open-flow nitrogen cryostat (Cosier & Glazer, 1986) operating at 120.0 (1) K.
Geometry. All e.s.d.'s (except the e.s.d. in the dihedral angle between two l.s. planes) are estimated using the full covariance matrix. The cell e.s.d.'s are taken into account individually in the estimation of e.s.d.'s in distances, angles and torsion angles; correlations between e.s.d.'s in cell parameters are only used when they are defined by crystal symmetry. An approximate (isotropic) treatment of cell e.s.d.'s is used for estimating e.s.d.'s involving l.s. planes.
Refinement. Refinement of *F*^2^ against ALL reflections. The weighted *R*-factor *wR* and goodness of fit *S* are based on *F*^2^, conventional *R*-factors *R* are based on *F*, with *F* set to zero for negative *F*^2^. The threshold expression of *F*^2^ > σ(*F*^2^) is used only for calculating *R*-factors(gt) *etc*. and is not relevant to the choice of reflections for refinement. *R*-factors based on *F*^2^ are statistically about twice as large as those based on *F*, and *R*- factors based on ALL data will be even larger.

### Fractional atomic coordinates and isotropic or equivalent isotropic displacement parameters (Å^2^)

**Table:**

	*x*	*y*	*z*	*U*_iso_*/*U*_eq_	
O1	0.38020 (7)	0.71239 (11)	0.65434 (5)	0.0236 (2)	
O2	0.40102 (7)	0.40284 (10)	0.49618 (5)	0.0226 (2)	
O3	0.10593 (7)	0.26403 (10)	0.35317 (5)	0.0237 (2)	
O4	−0.00667 (7)	0.42294 (10)	0.40878 (5)	0.0230 (2)	
N1	0.09228 (9)	1.16277 (13)	0.71073 (6)	0.0226 (3)	
H1N1	0.0289 (15)	1.1700 (18)	0.6971 (8)	0.028 (5)*	
H2N1	0.1136 (15)	1.197 (2)	0.7468 (9)	0.036 (5)*	
C1	0.23640 (9)	0.84035 (13)	0.65403 (6)	0.0165 (3)	
C2	0.28745 (9)	0.92608 (13)	0.69789 (6)	0.0169 (3)	
H2A	0.3552	0.9129	0.7131	0.020*	
C3	0.23973 (10)	1.02891 (13)	0.71892 (6)	0.0174 (3)	
H3A	0.2749	1.0829	0.7486	0.021*	
C4	0.13779 (10)	1.05267 (13)	0.69558 (6)	0.0171 (3)	
C5	0.08494 (9)	0.96364 (14)	0.65447 (6)	0.0183 (3)	
H5A	0.0166	0.9746	0.6407	0.022*	
C6	0.13318 (10)	0.86001 (14)	0.63425 (6)	0.0182 (3)	
H6A	0.0968	0.8020	0.6070	0.022*	
C7	0.29247 (10)	0.73650 (13)	0.63014 (6)	0.0177 (3)	
C8	0.24234 (10)	0.66396 (14)	0.57644 (6)	0.0201 (3)	
H8A	0.1762	0.6835	0.5598	0.024*	
C9	0.28853 (9)	0.57075 (13)	0.55064 (6)	0.0177 (3)	
H9A	0.3553	0.5549	0.5673	0.021*	
C10	0.24334 (9)	0.49221 (13)	0.49882 (6)	0.0163 (3)	
C11	0.30112 (9)	0.40495 (13)	0.47178 (6)	0.0174 (3)	
C12	0.25784 (10)	0.32686 (13)	0.42310 (6)	0.0185 (3)	
H12A	0.2973	0.2697	0.4059	0.022*	
C13	0.15546 (10)	0.33465 (13)	0.40038 (6)	0.0181 (3)	
C14	0.09645 (9)	0.42119 (13)	0.42698 (6)	0.0181 (3)	
C15	0.14011 (10)	0.49721 (13)	0.47490 (6)	0.0177 (3)	
H15A	0.1002	0.5538	0.4921	0.021*	
C16	0.46300 (10)	0.31421 (15)	0.47127 (7)	0.0227 (3)	
H16A	0.5310	0.3231	0.4922	0.034*	
H16B	0.4590	0.3324	0.4288	0.034*	
H16C	0.4406	0.2276	0.4760	0.034*	
C17	0.16178 (11)	0.17238 (15)	0.32512 (7)	0.0249 (3)	
H17A	0.1189	0.1338	0.2908	0.037*	
H17B	0.1877	0.1065	0.3540	0.037*	
H17C	0.2162	0.2155	0.3118	0.037*	
C18	−0.04143 (12)	0.49413 (19)	0.35457 (8)	0.0338 (4)	
H18A	−0.1129	0.5017	0.3482	0.051*	
H18B	−0.0228	0.4500	0.3207	0.051*	
H18C	−0.0120	0.5787	0.3582	0.051*	

### Atomic displacement parameters (Å^2^)

**Table:**

	*U*^11^	*U*^22^	*U*^33^	*U*^12^	*U*^13^	*U*^23^
O1	0.0160 (5)	0.0295 (6)	0.0228 (5)	0.0039 (4)	−0.0025 (4)	−0.0032 (4)
O2	0.0130 (4)	0.0288 (6)	0.0253 (5)	0.0038 (4)	0.0019 (4)	−0.0025 (4)
O3	0.0197 (5)	0.0270 (5)	0.0244 (5)	−0.0026 (4)	0.0043 (4)	−0.0092 (4)
O4	0.0132 (4)	0.0286 (6)	0.0263 (5)	−0.0023 (4)	0.0012 (4)	−0.0023 (4)
N1	0.0168 (6)	0.0277 (7)	0.0220 (6)	0.0043 (5)	0.0001 (4)	−0.0045 (5)
C1	0.0151 (6)	0.0176 (6)	0.0160 (6)	−0.0007 (5)	0.0009 (4)	0.0023 (5)
C2	0.0126 (5)	0.0202 (6)	0.0164 (6)	−0.0009 (5)	−0.0009 (4)	0.0036 (5)
C3	0.0157 (6)	0.0201 (6)	0.0151 (6)	−0.0029 (5)	−0.0004 (4)	0.0012 (5)
C4	0.0162 (6)	0.0200 (6)	0.0152 (6)	−0.0005 (5)	0.0035 (4)	0.0034 (5)
C5	0.0116 (5)	0.0230 (7)	0.0195 (6)	−0.0014 (5)	0.0003 (4)	0.0023 (5)
C6	0.0151 (6)	0.0206 (6)	0.0176 (6)	−0.0038 (5)	−0.0002 (4)	0.0006 (5)
C7	0.0160 (6)	0.0194 (6)	0.0170 (6)	−0.0002 (5)	0.0012 (5)	0.0022 (5)
C8	0.0151 (6)	0.0225 (7)	0.0203 (6)	0.0011 (5)	−0.0024 (5)	−0.0015 (5)
C9	0.0137 (5)	0.0220 (7)	0.0170 (6)	−0.0018 (5)	0.0019 (4)	0.0029 (5)
C10	0.0145 (6)	0.0184 (6)	0.0161 (6)	−0.0008 (5)	0.0034 (4)	0.0018 (5)
C11	0.0135 (5)	0.0202 (6)	0.0186 (6)	−0.0002 (5)	0.0034 (5)	0.0035 (5)
C12	0.0178 (6)	0.0186 (6)	0.0209 (6)	0.0013 (5)	0.0080 (5)	0.0005 (5)
C13	0.0190 (6)	0.0186 (6)	0.0174 (6)	−0.0038 (5)	0.0052 (5)	−0.0006 (5)
C14	0.0129 (6)	0.0208 (7)	0.0206 (6)	−0.0023 (5)	0.0033 (5)	0.0008 (5)
C15	0.0148 (6)	0.0187 (6)	0.0200 (6)	0.0003 (5)	0.0045 (5)	−0.0005 (5)
C16	0.0166 (6)	0.0260 (7)	0.0265 (7)	0.0058 (5)	0.0071 (5)	0.0040 (6)
C17	0.0281 (7)	0.0221 (7)	0.0260 (7)	−0.0023 (6)	0.0091 (6)	−0.0064 (6)
C18	0.0208 (7)	0.0432 (10)	0.0345 (9)	0.0005 (7)	−0.0023 (6)	0.0071 (7)

### Geometric parameters (Å, °)

**Table:**

O1—C7	1.2400 (16)	C8—C9	1.3407 (19)
O2—C11	1.3666 (15)	C8—H8A	0.9300
O2—C16	1.4293 (17)	C9—C10	1.4549 (18)
O3—C13	1.3557 (16)	C9—H9A	0.9300
O3—C17	1.4329 (17)	C10—C15	1.4071 (18)
O4—C14	1.3859 (15)	C10—C11	1.4076 (18)
O4—C18	1.4228 (19)	C11—C12	1.3953 (19)
N1—C4	1.3704 (18)	C12—C13	1.3927 (18)
N1—H1N1	0.862 (19)	C12—H12A	0.9300
N1—H2N1	0.88 (2)	C13—C14	1.4083 (19)
C1—C6	1.4060 (18)	C14—C15	1.3724 (19)
C1—C2	1.4064 (18)	C15—H15A	0.9300
C1—C7	1.4760 (19)	C16—H16A	0.9600
C2—C3	1.3758 (19)	C16—H16B	0.9600
C2—H2A	0.9300	C16—H16C	0.9600
C3—C4	1.4089 (18)	C17—H17A	0.9600
C3—H3A	0.9300	C17—H17B	0.9600
C4—C5	1.4016 (19)	C17—H17C	0.9600
C5—C6	1.378 (2)	C18—H18A	0.9600
C5—H5A	0.9300	C18—H18B	0.9600
C6—H6A	0.9300	C18—H18C	0.9600
C7—C8	1.4718 (19)		
			
C11—O2—C16	118.06 (11)	C11—C10—C9	121.02 (12)
C13—O3—C17	118.17 (11)	O2—C11—C12	123.00 (12)
C14—O4—C18	114.38 (11)	O2—C11—C10	115.65 (12)
C4—N1—H1N1	117.0 (13)	C12—C11—C10	121.35 (12)
C4—N1—H2N1	118.4 (13)	C13—C12—C11	119.84 (12)
H1N1—N1—H2N1	115.1 (18)	C13—C12—H12A	120.1
C6—C1—C2	117.47 (12)	C11—C12—H12A	120.1
C6—C1—C7	123.07 (12)	O3—C13—C12	124.72 (12)
C2—C1—C7	119.46 (11)	O3—C13—C14	115.69 (12)
C3—C2—C1	121.63 (12)	C12—C13—C14	119.59 (12)
C3—C2—H2A	119.2	C15—C14—O4	119.17 (12)
C1—C2—H2A	119.2	C15—C14—C13	119.88 (12)
C2—C3—C4	120.24 (12)	O4—C14—C13	120.72 (12)
C2—C3—H3A	119.9	C14—C15—C10	122.04 (12)
C4—C3—H3A	119.9	C14—C15—H15A	119.0
N1—C4—C5	120.65 (12)	C10—C15—H15A	119.0
N1—C4—C3	120.86 (12)	O2—C16—H16A	109.5
C5—C4—C3	118.44 (12)	O2—C16—H16B	109.5
C6—C5—C4	120.69 (12)	H16A—C16—H16B	109.5
C6—C5—H5A	119.7	O2—C16—H16C	109.5
C4—C5—H5A	119.7	H16A—C16—H16C	109.5
C5—C6—C1	121.26 (12)	H16B—C16—H16C	109.5
C5—C6—H6A	119.4	O3—C17—H17A	109.5
C1—C6—H6A	119.4	O3—C17—H17B	109.5
O1—C7—C8	120.93 (12)	H17A—C17—H17B	109.5
O1—C7—C1	120.63 (12)	O3—C17—H17C	109.5
C8—C7—C1	118.43 (11)	H17A—C17—H17C	109.5
C9—C8—C7	122.45 (12)	H17B—C17—H17C	109.5
C9—C8—H8A	118.8	O4—C18—H18A	109.5
C7—C8—H8A	118.8	O4—C18—H18B	109.5
C8—C9—C10	125.73 (12)	H18A—C18—H18B	109.5
C8—C9—H9A	117.1	O4—C18—H18C	109.5
C10—C9—H9A	117.1	H18A—C18—H18C	109.5
C15—C10—C11	117.31 (12)	H18B—C18—H18C	109.5
C15—C10—C9	121.64 (12)		
			
C6—C1—C2—C3	−2.81 (19)	C15—C10—C11—O2	179.96 (11)
C7—C1—C2—C3	176.14 (12)	C9—C10—C11—O2	−1.95 (18)
C1—C2—C3—C4	−1.5 (2)	C15—C10—C11—C12	0.21 (19)
C2—C3—C4—N1	−172.31 (13)	C9—C10—C11—C12	178.30 (12)
C2—C3—C4—C5	5.09 (19)	O2—C11—C12—C13	−179.69 (12)
N1—C4—C5—C6	173.09 (13)	C10—C11—C12—C13	0.0 (2)
C3—C4—C5—C6	−4.31 (19)	C17—O3—C13—C12	1.5 (2)
C4—C5—C6—C1	−0.1 (2)	C17—O3—C13—C14	−178.72 (12)
C2—C1—C6—C5	3.6 (2)	C11—C12—C13—O3	179.60 (13)
C7—C1—C6—C5	−175.30 (13)	C11—C12—C13—C14	−0.2 (2)
C6—C1—C7—O1	−170.62 (13)	C18—O4—C14—C15	104.12 (16)
C2—C1—C7—O1	10.5 (2)	C18—O4—C14—C13	−81.36 (17)
C6—C1—C7—C8	10.37 (19)	O3—C13—C14—C15	−179.72 (12)
C2—C1—C7—C8	−168.52 (12)	C12—C13—C14—C15	0.1 (2)
O1—C7—C8—C9	−1.0 (2)	O3—C13—C14—O4	5.79 (19)
C1—C7—C8—C9	177.98 (13)	C12—C13—C14—O4	−174.38 (12)
C7—C8—C9—C10	177.99 (13)	O4—C14—C15—C10	174.73 (12)
C8—C9—C10—C15	−7.9 (2)	C13—C14—C15—C10	0.2 (2)
C8—C9—C10—C11	174.10 (14)	C11—C10—C15—C14	−0.3 (2)
C16—O2—C11—C12	−1.45 (19)	C9—C10—C15—C14	−178.39 (13)
C16—O2—C11—C10	178.80 (12)		

### Hydrogen-bond geometry (Å, °)

**Table:**

*D*—H···*A*	*D*—H	H···*A*	*D*···*A*	*D*—H···*A*
N1—H1N1···O1^i^	0.86 (2)	2.12 (2)	2.9692 (16)	170.4 (17)
N1—H2N1···O1^ii^	0.88 (2)	2.21 (2)	3.0176 (17)	153.4 (19)

Symmetry codes: (i) *x*−1/2, *y*+1/2, *z*; (ii) −*x*+1/2, *y*+1/2, −*z*+3/2.
